# Radiomics and Radiogenomics in Preclinical Imaging on Murine Models: A Narrative Review

**DOI:** 10.3390/jpm13081204

**Published:** 2023-07-29

**Authors:** Serena Monti, Maria Elena Truppa, Sandra Albanese, Marcello Mancini

**Affiliations:** National Research Council, Institute of Biostructures and Bioimaging, 80145 Naples, Italy; serena.monti@ibb.cnr.it (S.M.); mariaelena.truppa@gmail.com (M.E.T.); direttore@ibb.cnr.it (M.M.)

**Keywords:** radiomics, radiogenomics, preclinical imaging, mouse model, murine model

## Abstract

Over the past decade, medical imaging technologies have become increasingly significant in both clinical and preclinical research, leading to a better understanding of disease processes and the development of new diagnostic and theranostic methods. Radiomic and radiogenomic approaches have furthered this progress by exploring the relationship between imaging characteristics, genomic information, and outcomes that qualitative interpretations may have overlooked, offering valuable insights for personalized medicine. Preclinical research allows for a controlled environment where various aspects of a pathology can be replicated in animal models, providing radiomic and radiogenomic approaches with the unique opportunity to investigate the causal connection between imaging and molecular factors. The aim of this review is to present the current state of the art in the application of radiomics and radiogenomics on murine models. This review will provide a brief description of relevant articles found in the literature with a discussion on the implications and potential translational relevance of these findings.

## 1. Introduction

Over the past decade, the use and significance of medical imaging technologies in clinical practice and research have undergone a significant expansion. Previously serving primarily as a diagnostic tool, imaging technologies have now assumed a central role within the context of individualized medicine.

Indeed, advancements in imaging modalities and techniques have enabled clinicians to gather a wealth of detailed information about a disease, giving imaging a crucial role in tailoring treatment strategies to the specific characteristics of each patient, in the assessment of treatment response, and in guiding interventions.

In this context, a field that shows great promise is radiomics. This term is used to describe the automated extraction of a high-dimensional set of quantitative imaging features for the definition of the imaging phenotype of a disease. This approach offers the possibility to analyze a pathology objectively, highlighting imaging-based characteristics which include shape, intensity, and texture features that may not be fully perceived by the human eye [1,2,3]. Radiomics offers a new approach to evidence-based medicine, showing great potential for its future usage in personalized medicine [4,5,6,7].

Alongside the advancement of imaging technology, the availability of large-scale genomic data has allowed researchers to explore the integration of imaging and genomic information to uncover underlying associations and predictive markers. Radiogenomics is a concept that explores the potential relationship between imaging, genomics, and clinical knowledge to mine quantitative imaging features and genomic data to identify patterns, relationships, and biomarkers that may be indicative of specific genetic alterations or clinical outcomes [8,9]. The data-driven nature of radiogenomics allows for the discovery of previously unnoticed associations or patterns that might be missed by conventional qualitative interpretations with the potential to uncover novel insights, facilitate personalized medicine, and guide clinical decision making in oncology and other fields.

The advancement in imaging technologies has also opened up unique opportunities in translational research. The development of high-resolution in vivo imaging technologies has paved the way in preclinical research for the use of medical images, which has greatly increased since the beginning of the 21st century. By using imaging to directly observe disease processes in live animal models, it is possible to non-invasively and repeatedly monitor disease progression or response to treatment, with significant advantages in preclinical research, e.g., a reduction in biological variability or the opportunity to acquire multimodal information without interfering with the biological processes under study. Furthermore, preclinical imaging techniques contribute to the refinement of and a reduction in animal use, enhancing the efficiency of preclinical research, in accordance with ethical considerations [10]. The introduction of such new imaging technologies, together with the development of new mouse models, has led to innovation in the study of new diagnostic and theranostic approaches [11].

The use of radiomic and radiogenomic approaches in the preclinical field represents a step forward in the understanding of the underlying mechanisms of a disease. In fact, the context of preclinical research with the use of animal models represents a breeding ground to investigate causality in the imaging–molecular connection, which in clinical research can be observed only in terms of correlation [12].

The aim of this review is to highlight the current state of the art on the use of radiomics and radiogenomics with regard to preclinical research in murine models. In particular, our purpose was to emphasize the added value of using radiomic and radiogenomic techniques in this controlled scenario. After a brief introduction on how the literature search was conducted, in the following sections a brief description of the articles found will be made, with increased attention on the application context and a short outline of technical details of radiomics and radiogenomics. The articles will be divided according to the used acquisition modality. Finally, considerations and possible implications in terms of translational relevance will be drawn.

## 2. Search Strategy

We used PubMed and Google Scholar to explore studies published in the last 10 years in the area of radiomics and radiogenomics in preclinical research. The search was implemented by using the following keywords: “radiomics”, “radiogenomics”, “preclinical imaging”, “preclinical model”, “animal model”, “murine model”. This narrative review is the outcome of the studies that have been thoroughly scrutinized by experts in the field (who are also the co-authors) to critically include or exclude them.

## 3. Applications in Computer Tomography

Computer tomography (CT) is an imaging technique based on the use of ionizing radiation. It is an anatomical imaging technique that measures X-ray attenuation by tissues and provides 3-dimensional tomographic data at microscopic spatial resolution of tissue attenuation with sub-second temporal resolution. This imaging modality is useful for the assessment of skeletal and lung abnormalities, heart function, and tumor diagnosis.

Radiomics was used to analyze high-resolution CT in order to obtain a support tool for the diagnosis and monitoring of systemic sclerosis and interstitial lung disease (SSc-ILD) in a cross-species approach, as reported in the study by Schniering et al. [13]. The authors, indeed, derived a prognostic quantitative radiomic risk score (qRISSc) for progression-free survival on two clinical cohorts, based on features computed on semi-automatically segmented lungs. The qRISSc was derived by two-step feature selection, including univariable Cox regression and cross-validated least absolute shrinkage and selection operator (LASSO) penalized regression. It comprised 26 radiomic features, 4 intensity features, 9 texture features, and 13 wavelet features. After having tested the reverse translation of the obtained qRISSc from patients to animal model, mice with bleomycin-induced pulmonary fibrosis [14] were used to investigate the correlation between the radiomic signature and biological features, including proteomic, histological, and gene expression data. The authors’ findings indicated that the qRISSc reflects an underlying fibrotic remodeling process in experimental ILD, suggesting that individuals with a high qRISSc score exhibit a dominant activation of fibrotic rather than inflammatory pathways.

A radiomic-based approach was also used to diagnose lung disease generated by polluted air [15]. For this experiment, BALBc/ByJ mice were divided into a control group and two exposed groups, one to sulfur dioxide (SO_2_), and the other to cigarette smoke combined with ozone. From the acquired CT images, the authors obtained a radiomic-based score, computed as the fit to two Gaussian curves of the fractal dimension of the chest at varying levels of attenuation, to distinguish between different air pollutants’ effects on the lungs. The obtained model was able to highlight different Gaussian parameters associated with patterns of respiratory pulmonary motion, increased mucus production, and increased presence of tissue microbubbles, providing a valuable tool to early diagnose chronic obstructive pulmonary disease-related lung and airway changes.

An oncological application of a CT-based radiomic approach is found in [16], a study on the differentiation of tumors based on lymphocyte burden. The authors used Rag2+/− and Rag 2−/− genetically modified mice (in which, respectively, B- and T-cells were mature or not) to generate sarcomas with or without lymphocytes [17,18,19]. After radiation therapy (RT), mice underwent dual-source hybrid spectral micro-CT with nanoparticle contrast media for vascular and tumor imaging. From a single acquisition, the authors obtained energy-integrating detector (EID)-CT, photon-counting detector (PCD)-CT at four different energies, and spectral decomposition maps of Iodine (I), Photoelectric effect (PE), and Compton Scattering (CS). The radiomic scores were computed in each of them separately, from a single semi-automated tumor segmentation (given the image spatial registration), using the Maximum Relevance Minimum Redundancy (MRMR) algorithm and a principal component analysis (PCA). The authors found that radiomics was able to distinguish between Rag2+/− and Rag2−/− mice, with the best performances reached by the PCD-CT based score, followed by the material map score and the EID-CT derived score, and the most informative contribution given by the texture and shape-based features found from the image wavelet transforms.

The causal relationship between genetic changes and the CT radiomic features was investigated by Panth et al. [20] in a genetic tumor model in which tumor microenvironmental characteristics like hypoxia change upon doxycycline administration. NMRI-nu/nu mice were injected with HCT116 doxycycline inducible GADD34 cells to obtain xenografts. Then, in order to observe the expression of the gene, doxycycline or placebo were administered; in addition, to evaluate the effects of treatment on image features, mice were treated with RT or received sham irradiation. Radiomic features were extracted from tumors segmented on CTs acquired at three time points (baseline, after RT, when tumor volume reached 500 mm^3^). After feature selection by means of the intra-class correlation coefficient (ICC), authors performed a Wilcoxon rank sum test to compare feature values between pairs of groups. Authors found that radiomic features were significantly different upon genetic change and RT monotherapy, but these features were not consistent between CT timepoints, with the feature selection step providing different features at each time point. However, these findings confirmed the hypothesis that tumor heterogeneity between gene-induced and non-induced tumors was reflected in the imaging features likely as a result of a phenotypic change, with dynamic changes occurring in the tumor and tumor microenvironment over time.

## 4. Applications in High-Frequency Ultrasound

Ultrasound (US) imaging is based on the interaction of sound waves with living tissues to produce images. In preclinical imaging, US systems use frequencies in the range of 20 to 50 MHz to obtain high spatial resolution and a penetration depth adequate to the anatomy of the animal model. High-Frequency US (HFUS) allows for the visualization of fine anatomical details without ionizing radiation, provides real-time functional information, and can be easily used for theranostic approach.

Bao et al. [21] applied a radiomic-like approach to multimode US imaging to detect early molecular changes in incomplete tumor ablation in animal models. The authors implanted HCT-26 colorectal adenoma in the liver of severe combined immunodeficiency (SCID) mice and then randomly divided them into a blank control group, a sham puncture group, and an incomplete laser ablation group. Shortly after, the subjects underwent contrast-enhanced US (CEUS), from which a radiomic model was established to differentiate between treated and untreated animals. For each lesion, a region of interest (ROI) around the tumor border was delineated and radiomic features from grayscale data, contrast-enhanced arterial phase data, and the whole course of CEUS data were extracted and selected using the LASSO regression model. The obtained results showed that the best-performing classification was obtained by the portal venous-phase model. Considering that incomplete ablation caused an increase in heat shock and apoptosis-related proteins, the authors’ findings showed that the radiomic classification can reveal early protein changes in tumors after incomplete ablation.

Another example of the application of radiomics to US includes the study by Theek et al. [22], in which the authors evaluated how radiomic analysis on CEUS images can help in the differentiation of three xenograft mouse tumor models. Radiomic features were computed on manually delineated ROI from B-mode images, together with functional parameters of the vasculature extracted from CEUS scans. Using a dedicated feature-selection algorithm, the obtained radiomic signature, resultantly composed by both morphological and functional parameters, confirmed that CEUS imaging can be used for computer-aided diagnosis.

## 5. Applications in Magnetic Resonance Imaging

Magnetic Resonance Imaging (MRI) is a non-ionizing 3D imaging technique that relies on the magnetic properties of tissues and their interactions with external magnetic fields. It typically utilizes the hydrogen nucleus from water molecules, providing images that give information on the number and the microenvironment of these nuclei within different tissues. MRI provides detailed morphological images with excellent contrast and spatial resolution, but also information about tissue composition, perfusion, oxygenation levels, tissue elasticity, metabolism, and even the detection of molecular probes or contrast agents.

Numerous studies on MRI can be found in the field of radiomics, but only one in the field of radiogenomics.

Zinn et al. [12] performed a co-clinical radiogenomic prediction and validation study. They collected glioblastoma patients’ data from The Cancer Genome Atlas (TCGA), The Cancer Imaging Archive (TCIA), and the Repository of Molecular Brain Neoplasia Data (REMBRANDT). After having semiautomatically segmented edema/invasion, active enhancing tumor, necrosis, and contralateral normal-appearing white matter regions, they extracted radiomic features and built a logistic regression model to classify patients into high- and low-Periostin-(POSTN)-expression status groups. To test the causality of the found radiogenomic association, the authors generated orthotopic xenograft models using two independent patient-derived glioma stem cells (GSC) lines upon validation of efficient short hairpin RNA (shRNA)-mediated POSTN knockdown. From the combination of the two cell lines and the control and POSTN-knockdown mouse tumors, four groups were obtained. They underwent MRI and xenograft tumors were analyzed by ex vivo immunohistochemistry to confirm protein expression levels. The authors identified specific radiomic features significantly distinguishing control and knockdown xenograft tumors and then applied this model to the clinical dataset, obtaining better prediction results than the model trained on the clinical dataset itself. These results demonstrated that gene expression drives a specific imaging feature phenotype, with gene expression profiles in patients that share significantly concordant radiomic features with their matching preclinical murine xenograft counterparts.

Radiomics was used on diffusion-weighted magnetic resonance imaging (DWI) and the derived apparent diffusion coefficient (ADC) to obtain predictions about RT response [23]. The biological significance of the ADC value was evaluated in a mouse model of prostatic tumor through the correlation between the ADC-based radiomic features and results obtained on biological samples excised to match as much as possible the MRI orientation plane. Each tumor was partially irradiated with a dose rate of 8 Gy/min, ADC features were extracted from treated and non-treated ROIs, and histological parameters were matched in order to correlate nuclear counts, nuclear sizes, nuclear spaces, cytoplasmic spaces, and extracellular spaces. Irradiated regions of the tumor showed higher values of almost all ADC radiomic features as well as, at histological analysis of the corresponding regions, increased extracellular space and nuclear size and decreased nuclear count. The voxel-by-voxel correlation of ADC maps with histological results highlighted a positive correlation with extracellular spaces and nuclear sizes and a negative correlation with nuclear counts, cytoplasmic space, and nuclear spaces. These results supported the benefit of the ADC analysis as a non-invasive method to characterize the heterogeneity of a tumor tissue, providing the evidence of the biological mechanisms by which RT may alter the ADC values measured by MRI.

Holbrook et al. [24], within the preclinical arm of a co-clinical trial, devised a new radiomic pipeline of MRI to segment sarcoma and evaluate RT effect and tumor recurrence. The authors generated a genetically engineered model of soft tissue sarcoma in p53 fl/fl mice. These subjects underwent MRI before and after RT. Radiomic analysis showed a change before and after treatment in the shape and in the texture of the tumor, suggesting that radiomics may be useful in detecting and monitoring the effects of high-dose radiation. In addition, the authors found that radiomic features in the peritumoral area could aid in determining the likelihood of primary tumor recurrence.

Radiomics was also used in the assessment of vaccine efficacy, as explained in the study by Eresen et al. [25], in which LSL-KrasG12D/+, LSL-Trp53R172H/+, Pdx-1-Cre (KPC) mouse model was used to understand the effect of dendritic cell vaccination on pancreatic ductal adenocarcinoma (PDAC). Mice were divided into treated and control groups and underwent weekly MRI. ROIs were drawn on the slice with maximal tumor diameter and radiomic features were computed, selected, and used to build a support machine classifier with a leave-one-out validation approach using an exhaustive search feature-selection method. The extracted radiomic model, comprising wavelet-based and gradient-based features, identified the treated tumors with increasing accuracy throughout the first three weeks of treatment. In addition, wavelet coefficients demonstrated a strong correlation with the survival of the KPC mice in the whole population and, together with gradient-based radiomic features, also with histological outcome, in particular fibrosis percentages and CK19 and Ki67 immunostains. These results demonstrated that MRI radiomic analysis can be used to early detect the immunological response to DC vaccine, as well as long-term outcomes in the KPC mouse model of PDAC.

A different abdominal tumor that is one of the major causes of death is colorectal cancer. Its symptoms are devastating, especially when accompanied by liver metastases. For this reason, predicting its occurrence is essential, as explained in the study of Becker et al. [26], which investigated whether MRI texture features show a correlation with tumor growth before metastasis can be diagnosed. In this study, a murine model was set up in C57BL6 males through intraportal injection of MC-38 tumor cells (or phosphate-buffered saline for the control group). The subjects underwent T2-weighted MRI at different time points, and an ROI encompassing a visible metastasis was contoured on the last time point image and copied into the previous acquisitions. Texture analysis was performed on these ROIs and the time variation of the features was analyzed in the two groups. The obtained results provided a proof of concept that texture analysis of MRI may have the potential to detect liver metastases at a sub-resolution level, before they become visible to the human eye.

Glioblastoma (GB) is one of the most aggressive brain tumors. Consequently, Núñez et al. [27] attempted to provide, in a preclinical study, an analytical pipeline that could be used in subsequent human studies. For this analysis, mice stereotactically injected with GL261 GB cells were divided in two groups (one treated with temozolomide and a control group) and analyzed with MRI and MR spectroscopic imaging (MRSI). The performed analysis led to controversial results in terms of stability or reproducibility, but highlighted the clear advantage in the combined use of radiomics from MRI and source separation from MRSI to obtain an intuitive, radiologist-friendly visualization tool that summarizes, in an anatomically recognizable representation, a complex combination of feature selection and classification, together with prediction certainty.

Muller et al. [28] examined a heterogeneous cohort of head and neck squamous cell carcinoma tumor-bearing mice (transplanted with the radioresistant SAS and radiosensitive UT-SCC-14 cell lines) to evaluate the correlation between quantitative features of MRI and tumor microenvironment (TME). To induce additional intra-phenotypic heterogeneity, the mice were subjected to fractionated irradiation with varying treatment regimens and beam modalities. After treatment, tumors were examined with contrast-enhanced MRI and histopathological evaluation. The tumors were segmented on post-contrast MRI and radiomic features were computed; similarly, microscopy image data were automatically processed to compute TME features. Risk models based on MRI, TME, and on their combination were developed and validated with a leave-one-out cross validation scheme. The authors obtained good tumor phenotype prediction performances both for prediction models based on MRI and on TME, with better results when using combined information. In addition, a moderate correlation was found between hypoxia-related TME features and texture-related MRI features, confirming the relevance of the detection of tumor hypoxia during curative radio-chemotherapy as a prognostic factor for treatment failure.

Roy et al. [29] performed a radiomic study on Triple-negative breast cancer (TNBC) patient-derived tumor xenograft (PDX) model in the context of a co-clinical trial whose primary objective was to identify robust T1-weighted and T2-weighted radiomic features for both preclinical and clinical imaging pipelines. The authors characterized the sensitivity of radiomic features to noise, image resolution, and tumor volume, which are some of the macroscopic differences between a clinical and preclinical MR imaging study. They also found that these factors significantly impact radiomic features, in particular higher-order features computed from textural matrices, and have to be taken into account in the design of a co-clinical trial with radiomic endpoints. Further, they implemented an algorithm to calibrate noise characteristics between preclinical and clinical images and identified the most volume-independent radiomic features.

A different application of radiomics in the same animals can be found in [30]. In this work, indeed, the authors devised a deep-learning based segmentation approach to accurately delineate the TNBC in the MR images of the PDX mice model. They finally extracted radiomic features from the segmented ROIs and used them to analyze the level of agreement between and within manual and automated contouring methods.

A radiomic application beyond the oncological field can be found in [31]. The authors’ aim was to identify the optimal radiomic model to predict liver fibrosis stage in Wistar rats from MR images. Rats were subcutaneously injected with a mixture of carbon tetrachloride and olive oil twice a week to induce liver fibrosis; the control group was injected with saline at the same volume. After MR scanning at multiple timepoints, rats were sacrificed and livers were fixed in formalin and subsequently underwent histology for the METAVIR scoring staging. Radiomic features were computed on liver ROI, avoiding major vessels and artifacts and, after feature selection by means of PCA and LASSO methods, different classification models were constructed for several classification tasks, demonstrating the feasibility of radiomic assessment of fibrosis from non-enhanced T1-weighted MRI.

In the area of neuroscience, Singh et al. [32] proposed a new approach combining radiomics with biologically sensitive neurite orientation dispersion and density index (NODDI) diffusion MR imaging, a methodology able to model compartment signals and capture microstructural information in biophysical locations, obtaining the extraneurite (ODI), intraneurite (NDI), and cerebrospinal fluid (CSF) space signals. The authors performed ex vivo imaging of rat brains and focalized their analysis on the amygdala, the hippocampus, and the corpus striatum regions (due to their biological relevance in autism spectrum disorder), computing first- and second-order radiomic features of ODI, NDI, and CSF signals. This combined approach showed satisfactory results in distinguishing differences between four genetically distinct rat models of autism spectrum disorder (ASD), i.e., Fmr1, Pten, Nrxn1, and Disc1. The ability of such an approach to subtype ASD models was also confirmed by an unsupervised clustering, suggesting that it may have the capacity to sensitively and specifically disambiguate the neurobiological heterogeneity present in the ASD population.

## 6. Applications in Positron Emission Tomography

Positron Emission Tomography (PET) is a well-established imaging technology that involves the utilization of a radiotracer, consisting of a biologically active molecule labeled with a positron-emitting radioisotope, whose biodistribution enables the visualization and quantification of specific biochemical and physiological processes. PET imaging provides functional and molecular information, high sensitivity, and the possibility of quantitative analysis, with possible fields of application ranging from oncology to neurology and cardiology, as far as infection and inflammation imaging.

In order to establish a valid model to predict therapeutic response in TNBC patient, Roy et al. [33] applied a radiomic approach to PET/CT images within their co-clinical trial. Radiomic features were collected from PET images with 18F-FDG both in clinical patients and in subtype-matched PDX xenograft mouse models, before and one time during Docetaxel/carboplatin treatment. The authors computed radiomic features in tumor ROI and identified the most robust radiomic features in terms of reproducibility, cross-correlation, and dependency on tumor volume. Then, they extracted two models to predict (on the basis of pre-treatment features) or assess (on the basis of the feature change between time points) response to Docetaxel/carboplatin therapy. The obtained machine learning radiomic signatures, optimized for PDX, and comprising four features from first- and second-order groups, was then applied in the clinical arm to evaluate therapeutic responses, resulting in a more effective predicting/assessing response to therapy than the common standard uptake volume metrics alone.

In the study by Benfante et al. [34], radiomics on PET imaging was used to evaluate the biodistribution of a new [64Cu] chelator. Three groups of female Balb/C nude mice were injected with this new radio-nuclide and underwent PET imaging, each at a different time point. After a normalization step to an atlas and automated segmentation of heart, bladder, stomach, spleen, liver, kidneys, and lungs, radiomic features from these organs were automatically extracted from SUV images and the results at different time points were compared, highlighting bladder and liver as the regions where most of the features showed significant variations among the three groups. These results made radiomics a good candidate for objective observation of biodistribution, opening the way towards a decision-support system in the context of new radiopharmaceutical studies.

## 7. Discussion

In the past decade, medical imaging technologies have expanded their use and significance in clinical and preclinical research, leading to increased understanding of disease processes and the development of new diagnostic and theranostic approaches. With the development of radiomic and radiogenomic approaches, which explore the imaging phenotype and its relationship with a generic given outcome and the genomic information, respectively, new associations and patterns that might be missed by qualitative interpretations have been highlighted, providing new insights for personalized medicine and clinical decision making. The use of radiomic and radiogenomic approaches in preclinical research contributes to understanding the underlying mechanisms of diseases, in a controlled context in which, in principle, different aspects of the pathology under investigation can be reproduced in animal models. One example may be the use of PDXs that, originating from human cells, are nowadays the oncological model of choice. The use of PDX avoids the inoculation of cells grown in vitro and derived from cell cultures, exploiting instead those derived from human tumors. This approach allows closer reproduction of human cancer in terms of histological characteristics and therapeutic response and, to some degree, is able to mimic its genetic heterogeneity. In addition, PDXs better depict the biological processes associated with metastatic spread and progression, representing a valid model for the clinical translatability of radiomic model developed in preclinical research.

If on one side, the translation of radiomics and radiogenomics between clinical and preclinical applications seems fairly straightforward because of the use of imaging and genetic techniques that are widely available in both contexts, on the other side a substantial difference exists between them. It lies, above all for the imaging aspects, in the size of the sample to be analyzed, because the analyzed ROIs (or VOIs) are much smaller in mice compared to humans. This implies that, to collect a similar amount of information, a completely different voxel size and resolution are needed, which in turn means a different sensitivity of radiomic features to noise and region volume.

In addition to technical considerations that are strictly related to these methodologies, the biological aspects implicitly related to the difference between animal models and human diseases have to be considered. For example, oncological models are generated using NOD SCID mice (immunocompromised strain) to allow the engraftment and growth of cancer cells. The use of immunocompromised mice limits their use in immunotherapy studies, but animal models of cancer remain the best solution, for example, to evaluate anti-cancer therapeutic efficacy, because cell cultures are not able to reproduce the complexity of human tumor microenvironment. Indeed, the possibility to test a drug’s efficacy/toxicity in a preclinical setting favors its success rate in a clinical setting [35].

In this review, several works have been described, the majority belonging to the field of radiomics, with just two contributions in the context of radiogenomics. The most used imaging modality in these preclinical studies is the MRI, followed by CT, PET, and US. Oncology is at the forefront among the found applications of radiomics and radiogenomics, with studies that range from the classification of a specific genotype, to the prediction of a treatment response, passing through more technical papers implementing optimal processing pipeline for translational research. However, some applications have also been found outside of oncology, in the fields of lung and liver diseases and neurological disorders.

Nevertheless, from this complex and multi-faceted scenario, some important take-home messages can be drawn. The most important goal of translational research is to develop methods in animal models which may later be used in the clinic. For radiomic/radiogenomic studies, this means first the usability of the algorithm in clinical practice. This implies numerous technical considerations, i.e., the differences in scales, resolution, and image quality between clinical and preclinical imaging, which could be properly addressed, for example, by well-designed co-clinical studies, using PDXs as co-clinical platforms. Despite some complexity, a wise use of radiomics and radiogenomics in preclinical imaging could really bridge the gap between the underlying mechanisms of a disease and its manifestation. Indeed, well-characterized and representative animal models would be highly valuable for testing specific hypotheses related to studying connections with pathophysiology. Only such an approach establishing the causality between the underlying molecular mechanism of a disease and its imaging phenotype would pave the way for the clinical acceptance of radiomics and radiogenomics.

## Data Availability

No new data were created or analyzed in this study. Data sharing is not applicable to this article.
